# An Evolutionary Model of “Sexual Conflict” Over Women's Age at Marriage: Implications for Child Mortality and Undernutrition

**DOI:** 10.3389/fpubh.2022.653433

**Published:** 2022-06-17

**Authors:** Jonathan C. K. Wells

**Affiliations:** Childhood Nutrition Research Centre, Population Policy and Practice Research and Teaching Department, UCL Great Ormond Street Institute of Child Health, London, United Kingdom

**Keywords:** child marriage, evolutionary theory, reproductive fitness, sexual conflict, maternal and child health, child undernutrition, public health intervention, education

## Abstract

**Background:**

Early women's marriage is associated with adverse outcomes for mothers and their offspring, including reduced human capital and increased child undernutrition and mortality. Despite preventive efforts, it remains common in many populations and is often favored by cultural norms. A key question is why it remains common, given such penalties. Using an evolutionary perspective, a simple mathematical model was developed to explore women's optimal marriage age under different circumstances, if the sole aim were to maximize maternal or paternal lifetime reproductive fitness (surviving offspring).

**Methods:**

The model was based on several assumptions, supported by empirical evidence, regarding relationships between women's marital age and parental and offspring outcomes. It assumes that later marriage promotes women's autonomy, enhancing control over fertility and childcare, but increases paternity uncertainty. Given these assumptions, optimal marriage ages for maximizing maternal and paternal fitness were calculated. The basic model was then used to simulate environmental changes or public health interventions, including shifts in child mortality, suppression of women's autonomy, or promoting women's contraception or education.

**Results:**

In the basic model, paternal fitness is maximized at lower women's marriage age than is maternal fitness, with the paternal optimum worsening child undernutrition and mortality. A family planning intervention delays marriage age and reduces child mortality and undernutrition, at a cost to paternal but not maternal fitness. Reductions in child mortality favor earlier marriage but increase child undernutrition, whereas ecological shocks that increase child mortality favor later marriage but reduce fitness of both parents. An education intervention favors later marriage and reduces child mortality and undernutrition, but at a cost to paternal fitness. Efforts to suppress maternal autonomy substantially increase fitness of both parents, but only if other members of the household provide compensatory childcare.

**Conclusion:**

Early women's marriage maximizes paternal fitness despite relatively high child mortality and undernutrition, by increasing fertility and reducing paternity uncertainty. This tension between the sexes over the optimal marriage age is sensitive to ecological stresses or interventions. Education interventions seem most likely to improve maternal and child outcomes, but may be resisted by males and their kin as they may reduce paternal fitness.

## Introduction

The high prevalence of child undernutrition in low- and middle-income countries is widely attributed to poverty, food insecurity, exposure to infectious diseases and other markers of social inequality. These multiple and interacting stresses have long been approached through a conceptual model first presented by the international organization UNICEF in 1990 (1). In this model, “immediate causes” include inadequate dietary intake and high infection rates; “underlying causes” include insufficient access to food, inadequate health infrastructure, poor care and feeding practices; while “basic causes” include the lack of financial and socio-economic resources available to households (e.g., education and employment) and inadequate political will (1). Recognizing the multifactorial pathway of risk, many types of intervention have been developed and tested, with varying degrees of success (2).

Much attention has focused on improving nutritional supply, targeting infant and young child feeding (IYCF) through promoting breast-feeding and appropriate complementary feeding, or providing micronutrient supplementation to pregnant mothers and young infants (2). Another focus has been efforts to improve “Water, Sanitation and Hygiene”, known as WASH interventions. Recently, three cluster-randomized trials in Kenya, Zimbabwe, and Bangladesh targeted both WASH and IYCF. Although poor sanitation was a strong predictor of child undernutrition (stunting) at baseline, all three trials found a small but significant benefit of IYCF, increasing height by 0.13–0.25 z-scores over 1.5–2 years, whereas there were no detectable benefits of WASH (3).

Beyond direct efforts to prevent maternal and child undernutrition, the UNICEF model indicates that more distal factors should also be targeted. It might be assumed that general economic and agricultural growth is key to improving populations' nutrition status, but the effects are disputed, and some analyses find weak or no effects (4, 5). This may be because such efforts fail to address the structural factors that underlie inequitable resource allocation within countries (6–9).

In this context there is increasing interest in the agency of the mother, represented by traits such as maternal education and autonomy. The UNICEF model certainly recognizes the key role of mothers in infant and child nutrition (1), but although many of the causes of malnutrition act *via* constraints on the mother, the relationship between women's wider status in society and child undernutrition has only recently become a focus of attention. Maternal education and empowerment may benefit child nutrition through their impact on factors such as women's control of their time, household income and resources, and on their mental health, confidence, and self-esteem (10, 11). A review by Smith and Haddad (9) found that women's education and empowerment and gender equality are among the key drivers of past reductions in stunting. Consistent with this, across 96 countries the “Gender Inequality Index” [a composite index of women's disadvantage in reproductive health, empowerment, and labor market participation (12)] was associated with rates of low birth weight, stunting, wasting, and mortality in children below 5 years of age, even after adjusting for countries' gross domestic product (13).

While many aspects of women's status merit attention, this study focuses on variability in women's age at marriage as a composite marker of education, autonomy, and empowerment.

### Early Marriage in Women

According to the UN, marriage before age 18 years represents a fundamental violation of human rights (14), yet in many countries, it remains common for marriage to take place before this threshold (15). In Nepal, for example, 40% of women aged 20–24 years married <18 years in 2016, despite a legal minimum age of 20 years, or 18 with parental consent (16). Furthermore, such national averages may conceal substantial rural/urban and socio-economic variation.

Early marriage is systematically associated with less education among women (17–19). In lowland Nepal, for example, an analysis of ~6,400 mothers aged 23–30 years participating in a cluster-randomized trial found that over three quarters had not attended school at all, and that only those who had stayed in school until secondary education (~18 years) were likely to marry above the legal age of 18 years (16).

Many studies have linked early marriage with an earlier onset of reproduction and a faster rate of childbearing, mediated by lack of opportunities to control fertility. Studies in South Asia in particular have linked early marriage with lower age at first birth, higher fertility and lower use of contraceptives (20–25). This elevated fertility reflects pressure from other members of the marital household on the young bride to abstain from contraception and produce offspring early (26). Analyzing data on women aged 35–49 years from 15 countries, Onagoruwa and Wodon (27) estimated that early marriage was associated with substantially higher fertility, and that ending child marriage would reduce total fertility by between 0.24 and 1.06 offspring per woman.

Importantly, in both South Asia and East Africa, early marriage of women has been identified as a strong risk factor for child stunting (28–30). A number of independent pathways may contribute. Early marriage predisposes to early age at first pregnancy, which is a risk factor for low birth weight and child undernutrition (31). In lowland Nepal, both early marriage and early age at first pregnancy have been independently associated with shorter final maternal height (32). Mothers who marry early may also experience higher levels of psychosocial stress due to their low position in the marital household hierarchy (33), and this stress may undermine child growth through impairing both placental nutrition and lactation (34). Reduced maternal autonomy, such as over access to money, the freedom to go to markets, or the opportunity to take children for medical care, has also been linked to child undernutrition (35, 36).

**Figure 1:**
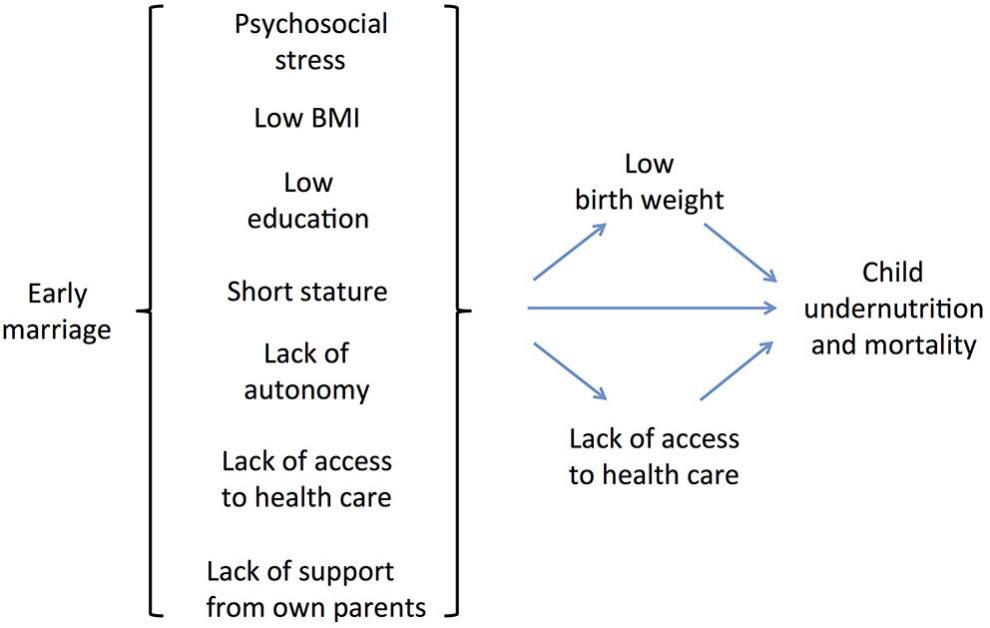
Conceptual model of the pathways linking women's early marriage to child undernutrition and mortality.

Overall, therefore, early marriage may contribute to child undernutrition through multiple pathways, including lack of education, lack of autonomy, lack of family support, early pregnancy, and high fertility (Figure 1). In this context, early marriage represents a cultural/institutional gateway that directs women along the pathway toward under-age childbearing and its adverse consequences. More broadly, because of its fundamental link with lack of education and low autonomy, the practice of under-age marriage is central to the propagation of societal gender inequality across generations (17, 37). For these reasons, early marriage can be considered a major issue in the context of health and human capital (17).

Despite the health and human capital penalties associated with early women's marriage, and despite extensive global efforts to change the practice, it remains common, particularly in countries with high levels of broader gender inequality. This prompts consideration of early marriage from an evolutionary perspective. Beyond the ethical and moral issues of maternal wellbeing, child morbidity and mortality are detrimental to the reproductive (genetic) fitness of both parents, defined as the number of surviving offspring produced who may carry the parents' genes on to future generations. Why should a cultural practice that harms the fitness of both parents persist?

### An Evolutionary Perspective on Women's Marriage Age

A key insight from evolutionary biology is that natural selection has shaped organisms to maximize genetic fitness over and above other outcomes such as health, optimal function or longevity (38, 39). Patterns of behavior that promote the production of viable offspring will, all other things being equal, spread in populations at the expense of competing behaviors associated with fewer offspring. In the context of human marriage patterns, several issues merit particular attention.

First, mammalian males and females are predicted to maximize their genetic fitness in different ways, due to their contrasting roles in the process of reproduction. Through placental nutrition and lactation, females play the dominant role in directly nourishing the offspring in early life, and their genetic fitness is constrained by their capacity to invest resources in successive offspring. For males, in contrast, the primary constraint on genetic fitness is the number of offspring that they can sire, for which the limiting factor is mating opportunities (40, 41). This perspective can be developed further, to understand tension between male and female kin over reproductive behaviors, such as the optimal age at which women should start to produce the offspring of a given male-female pairing if the aim were to maximize the reproductive fitness of each parent.

Second, sexual conflict over reproduction is exacerbated through a fundamental difference between the parents over parental “confidence.” Whereas mothers can have total confidence that they share 50% of their genes with each offspring, husbands cannot guarantee that they are the father of any offspring. The resulting “paternity uncertainty” is ultimately the source of male efforts to “monitor” the behavior of their mates/partners (42, 43). In humans, concern over paternity certainty may apply especially to patriarchal patrilocal societies, where the transfer of material property down the male line will only benefit the husband's genes if the offspring of his wife are indeed his own (44).

This issue is very relevant to the previous point regarding how the two parents maximize genetic fitness. In a polygamous society, males may legitimately increase fitness by reproducing with more than one female, but in a society where the social norm is monogamy, as in most South Asian societies, males can only legitimately maximize their own fitness through the fitness of their wife, conditional on their being the father of the offspring produced. Although men might also covertly increase their fitness through extra-marital mating, they cannot steer economic resources to such illegitimate offspring.

Third, beyond material resources pertaining to households such as agricultural land, housing quality and financial income, investment in offspring is also a function of “maternal capital,” a generic term derived from the concept of embodied capital (45) that refers to a wide range of maternal phenotypic traits (e.g., nutritional status, social support network, economic resources, empowerment, and education) that promote maternal investment in offspring (46). Among mammals in general, maternal physiological capital underpins nutritional investment during pregnancy and lactation (47). This period is of particular importance for long-term health of the offspring, due to fetal life and infancy representing “critical windows” during which vital organs and physiological traits develop (48, 49). On this basis, any tension between the two sexes over reproductive behavior in general may extend specifically to sexual conflict over strategies for the investment of maternal capital. Importantly, the strategies for investing maternal capital that may maximize the fitness benefits (i.e., increasing fertility) may be very different to those that maximize maternal and child health outcomes (47, 50).

If early marriage systematically reduces the health and human capital outcomes of both mothers and offspring, one might assume that fathers would themselves gain fitness benefits by marrying women later in adolescence. High rates of child malnutrition and mortality among the offspring of early-marrying women must inevitably represent a direct fitness cost to the fathers of these offspring. The same penalty could be inflicted on his grandchildren in the next generation, through the early marriage of his daughters. Higher rates of maternal mortality also “write down” any economic investment of men associated with their marriages, though the opposite scenario applies when dowry is contributed by the wife, as further marriages bring additional dowry. At first sight, therefore, the tendency for males to marry very young brides may seem counter-intuitive.

To shed light on to this paradox, I develop a mathematical model predicting how males and females may optimize genetic fitness according to both their own phenotypic traits and those of their partners. The model takes into account the multiple associations of women's age at marriage with the behavioral and somatic traits summarized above. I further address how these traits may be variably associated with paternity certainty. I use this model to show how contrasting certainty over parentage between males and females can drive contrasting optimal ages of women's marriage, with selection generally favoring a later age of marriage to maximize female fitness compared to the age that maximizes male fitness. I then show how the outcomes of each parent may change in different ways (i.e., shifting the optimal age at marriage for each sex) by simulating changes in socio-ecological conditions.

## Methods

The model is specifically intended to shed light on marriage practices in patriarchal societies (such as those typical of South Asia) where monogamy and early marriage are the norm, and where husbands' domination of their wives manifests through diverse forms of power and gender privilege (51). In this global region, rates of maternal and child mortality were historically very high, and mortality and malnutrition still remain major public health issues (52). The rationale for the evolutionary model is set out in detail in Box 1.

Box 1 **Developing an evolutionary model of sexual conflict over marriage age.**

Although earlier marriage is currently seen as a risk factor for adverse maternal and child health outcomes in traditional South Asian societies (28–30), it may also have emerged in the past as a solution to the very same challenges—a strategy for family decisions relating to demography, given the importance of producing children in patrilocal patrilineal societies (44).

**Figure 2:**
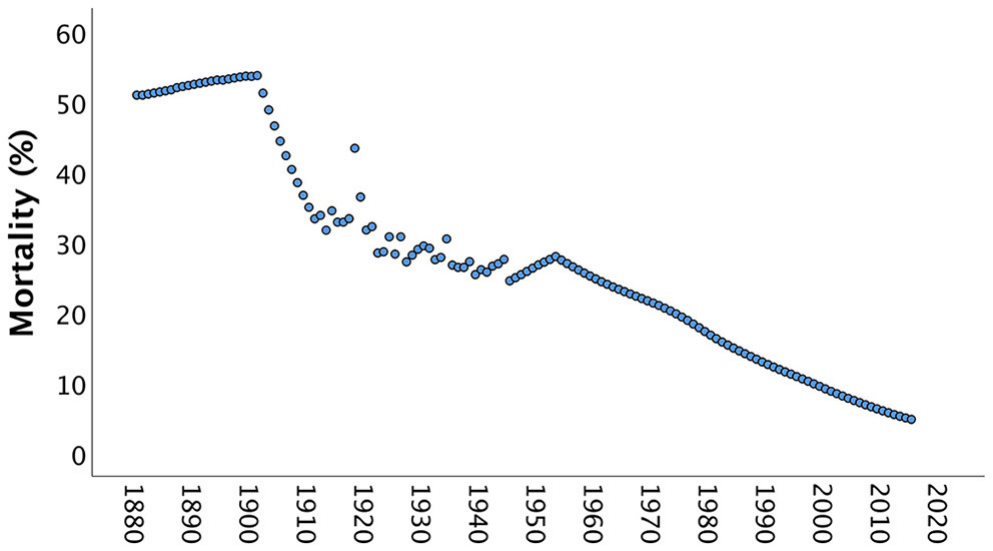
Mortality rates of children aged below 5 years in India between 1880 and 2015. Until the early 20th century, over half of all children born died, and it was only from the 1960s onwards that mortality rates dropped below 1 in 3 children. Data from https://www.gapminder.org/data/documentation/gd005/ (accessed April 24, 2020).

In the late 19th century, it is estimated that over half of all children born in India died before they reached 5 years of age (Figure 2). Families needed to compensate for these deaths with high fertility rates, and this pressure would be exacerbated by the importance attached to producing sons to maintain the family farm and name, and the greater susceptibility of sons to malnutrition and mortality in early life (53).

Producing offspring would thus have been a central concern in these societies, and this in turn would have favored earlier marriage of women in order to initiate the reproductive career during adolescence. Thus, marriage decisions would have aligned closely with the evolutionary goal of maximizing reproductive fitness.

As Figure 2 shows, child mortality levels fell across the 20th century, but nevertheless remained at around one third of births in the mid-century, and at 1 in 10 by the start of the 21st century. Social norms are often strongly enforced by older generations, and contemporary older adults grew up in eras with higher child mortality rates. Therefore, even if contemporary child survival is much improved, marriage decisions may be made under the influence of the experience of past generations.

Beyond child mortality, maternal mortality (deaths of associated directly with reproduction) in India was twice as high in the 1960s as the 1990s (54), and remains a major issue in contemporary South Asian populations. In poorer communities, 1 in every 6 mothers may die from maternal mortality, while women are also subject to additional mortality risks such as infection (55). High rates of death among women may likewise have favored the recruitment of younger wives, to initiate reproduction earlier.

A similar issue relates to the management of the risk of infidelity. In traditional South Asian societies, young women's behavior is often closely monitored, limiting interaction with men outside the family. On this basis, infidelity might appear a negligible risk. However, in other societies without such monitoring, extra-pair mating can be very common (56), hence the current practices and low infidelity levels in South Asian societies can be considered to go together. It should also be noted that beyond any voluntary extra-pair mating by women, non-consensual interactions (rape) might be a greater risk, and may also favor monitoring (57, 58). From an evolutionary perspective, men are highly motivated to minimize infidelity because it would lead to them investing economic resources in offspring with whom they share no genes. Although every husband could in theory seek additional offspring outside his marriage, he would have minimal influence over allocating resources to them.

Overall, efforts to maximize family size and guarantee paternity of offspring may therefore be considered rational from both evolutionary and cultural perspectives in environments where child mortality risk is high, and sons remain in the natal household to carry on the family farm.

For these reasons, a model based on evolutionary theory, exploring how age at marriage might maximize the fitness of the two parents, may shed insight into the persistence of early marriage over time. The importance of the issue of “infidelity” is that any reduction in paternity certainty drives men to favor an earlier commencement of their wife's reproductive career, in order to compensate for the “offspring lost to other fathers” by producing more offspring overall.

The basic form of the model is to generate several equations that describe assumed associations of marriage age with other variables, reflecting what has been described in the literature. By assuming that later age at marriage impacts maternal traits such as autonomy, inter-birth interval, risk of infidelity and nutritional status of the offspring, I can predict, for any age of marriage, both (a) how many offspring will be produced, and (b) how many will survive.

Having constructed the model by integrating all the equations, I can then vary one variable at a time while holding the others constant. This highlights the effect of each trait when considered in combination with the others. The primary outcome of the model is the *reproductive fitness* of each parent, calculated as the number of surviving offspring (all those born, minus those predicted to have died by 5 years). For each sex, individuals are being compared in these models against others of the same sex whose marriage occurred when the wife was younger or older.

Importantly, the two parents will not have identical values for reproductive fitness if there is any degree of paternity uncertainty. Any difference in the optimal age at marriage of mothers and fathers is then considered to indicate “*sexual tension*” over the optimal marriage age, i.e., the “goal” of maximizing fitness pulls males and females toward different ideal ages for the woman at marriage. Although data on the risks of child malnutrition and mortality are fed into the model to calculate fitness, once the values for optimal marriage age have been determined it is informative to see what specific levels of childhood malnutrition and mortality underlie those results for each parent. For example, in any specific setting, males might maximize their fitness not only through earlier marriage age compared to females, but also at a higher cost in absolute terms of child mortality and malnutrition.

The aim of this modeling is not to predict empirical values in any specific setting, but rather to illustrate how the fitness of each parent responds differently to modifiable aspects of cultural behavior and living conditions. Many mathematical models use arbitrary values for inputs (e.g., ranging between 0 and 1), which consequently produce findings that are not easy to interpret in relation to the literature, meaning that the implications for policy are also difficult to communicate. For this reason, I have selected numerical parameter values such that the optimal age at marriage identified in the modeling may be before or after 18 years, the most widespread legal definition of early marriage. These values are for illustrative purposes only, to provide a useful way to illustrate the consequences of simulated interventions. The approach is based on several specific assumptions, as follows.

### Assumptions

First, I assume that at some point, all women are married and subsequently produce offspring at a variable rate, which is broadly consistent with empirical evidence (59). Since I am interested in how age at marriage impacts life opportunities and physical traits of mothers, I assume for simplicity that the age of men's marriage has no impact on any of the traits or outcomes explored here. The aim is thus to explore the consequences of the timing and scheduling of women's marriage and reproduction on the fitness of each parent.

Second, I assume that delaying marriage, which is usually associated with staying in school during adolescence up to ~18 years (16), leads to greater overall female autonomy, as reported in diverse settings (60, 61). Underlying mechanisms may extend beyond what is learnt in school, and could also include broader benefits such as greater self-esteem, a larger social network that could provide social support during adult life, and having longer to acquire skills and expertise informally in the natal home (62). Exactly how women's autonomy increases with age prior to marriage is unclear, however here I assume a linear association with age, based on data on the association of women's marriage age with the average number of years of education (16).

Collectively, women's autonomy may relate to a number of different domains of female life. These domains may include decision-making in the household and around childcare, and control of the family food and monetary budget. All other things being equal, it is not intuitive that males would not value such autonomy, if it translated into better quality childcare for their own offspring. However, female autonomy is also associated with greater control over fertility and contraception (63, 64), which may be more antagonistic to paternal interests. Moreover, more education and autonomy and a larger social network might also increase women's opportunities for infidelity, thus reducing paternity certainty. Women remaining unmarried for longer may have greater opportunity to meet different men, and their greater autonomy may give them greater confidence to resist the authority and constraints of their husbands. My third assumption is therefore that earlier age at marriage curtails women's education and autonomy in linear dose-response manner, and that this is a strategy explicitly favored by males.

Fourth, I assume that early-onset of reproduction reduces maternal linear growth during adolescence (32, 65, 66). In turn, this has implications for child undernutrition in the next generation, as height of the mother at the level of the population predicts both birth weight and the risk of stunting in the offspring (67, 68), while other studies suggest that shorter women may also have lower household autonomy (69). On this basis, early age marriage is predicted to generate long-term penalties on maternal fitness mediated by shorter maternal stature. A previous mathematical model estimated an optimum height for women, reflecting a trade-off between the duration of growth and the rate of infant mortality (70). I therefore assume a linear dose-response association between women's marriage and maternal stature, as demonstrated in a study in lowland rural Nepal (32).

Fifth, following from the associations described above, earlier women's age at marriage would be expected to increase the likelihood of undernutrition among the offspring. Although child undernutrition has historically been conceptualized as comprising two distinct problems—severe underweight (termed “wasting”) and inadequate linear growth (termed “stunting”), recent work has highlighted that the two forms share many common causes, including low birth weight, and that each form is a risk factor for the other developing (71, 72). Of particular relevance here, an analysis of children from five birth cohorts in low and middle-income countries found that early maternal age at reproduction was associated with lower birth weight and higher risk of child stunting (31). Building on this, and on literature showing that early marriage increases psychosocial stress which can impair both fetal and infant growth (73, 74), I assume a dose-response declining association of age at marriage with the likelihood of delivering low birth weight offspring. I further assume an inverse dose-response linear association of age at marriage with the risk of undernutrition (stunting) worsening in post-natal life, as reduced autonomy limits the mother in making decisions over childhood nutrition of medical care.

Sixth, greater age at marriage is assumed to increase the inter-birth interval, since as discussed above, more educated and more autonomous women are likely to have greater control over fertility.

A seventh assumption is that earlier age at marriage may increase the risk of maternal mortality. Aside from infectious diseases that may strike mothers randomly, the risk of maternal mortality is specifically associated with each reproductive event. However, while a survey on maternal and newborn health across 29 countries by the World Health Organization found that, compared to mothers aged 20–24 years, adolescents under the age of 16 years had higher risks of cesarean section, eclampsia, and uterine and systemic infections (75), a comprehensive analysis of data from 144 countries and territories found that adolescent mothers had only slightly greater mortality risk compared to mothers aged 20–24 years (76). On this basis, maternal mortality risk was not incorporated in the basic model, instead I investigated how the results would change if mortality were to increase with decreasing maternal age. For simplicity, I assume that males themselves have zero mortality risk, and that if their wife dies at any point, they do not remarry and therefore bear fitness penalties matched to their first wife's.

### Modeling

**Figure 3:**
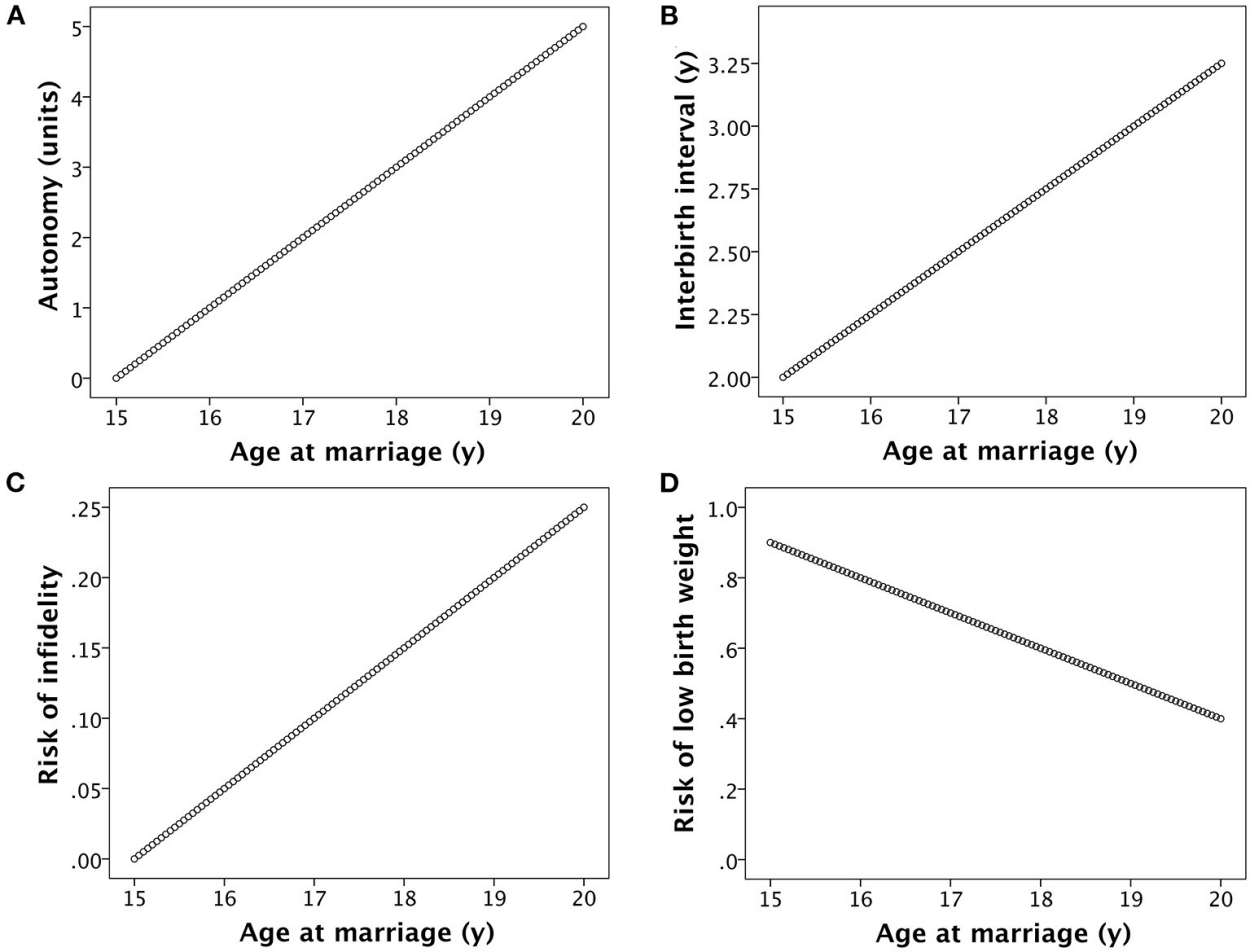
Basic assumptions in the model. **(A)** Women's autonomy increases in association with age at marriage, due to a combination of greater formal education and greater informal investment in skills and competencies by members of the natal household. **(B)** The inter-birth interval increases in association with women's age at marriage, due to greater autonomy providing increased control over contraception and fertility. **(C)** The risk of women's infidelity increases in association with their age at marriage, mediated by the development of a large social network and greater autonomy. **(D)** The risk of low birth weight declines with increasing women's age at marriage, mediated by factors such as greater autonomy over decisions related to child care and longer inter-birth intervals.

Each of the associations for the basic model may be illustrated graphically (Figure 3), and also described by mathematical functions. Assuming that all women produce their first offspring 1 year after their marriage, that the duration of the inter-birth interval increases in linear association with marital age (due to the underlying associations of marital age with female education and autonomy), and that women reproduce until 35 years, I can predict life-time fertility according to a woman's age of marriage. Figure 3 illustrates the assumptions in the basic model for the marital age range 15–20 years, however wider age ranges are investigated when simulating changes in socio-ecological conditions as discussed below.

Total fertility (TF) is calculated as follows:


(1) $$\begin{array}{l} \text{TF }=\;\left(\text{k}1-\text{AFP}\right)/\text{IBI} \end{array}$$

where AFP is age at first pregnancy, IBI is the duration of the inter-birth interval, and k_1_ is 35 according to the assumptions above. I assume that AFP is equal to marriage age (M) + 1 year, and that IBI increases linearly with age at marriage, from a value of 2 for marriage at 15 years to 3.25 at 20 years (Figure 3B). Thus


(2) $$\begin{array}{l} \text{IBI }=\;2\;+\;\left(\left(\text{M}-15\right)\;*\;0.25\right) \end{array}$$

I assume that the risk of low birth weight (LBW) decreases linearly with maternal age, and that this risk shapes the likelihood of child mortality. Given that the optimum birth weight for survival is between +1 and +2 z-scores (~3.8 kg) (77), I use for convenience a cut-off of 3 kg to define low birth weight rather than the conventional value of 2.5 kg. Mean birth weight in rural low-income South Asian societies where early marriage is common can be as low as ~2.7 kg (78), while birth weight also increases substantially with increasing maternal age (31, 78). I thus assume that the likelihood of producing an offspring with low birth weight decreases linearly from 90% for marriage at 15 years to 40% for marriage at 20 years (Figure 3D).


(3) $$\begin{array}{l} \text{LBW }=\;0.9\;-\;\left(\left(\text{M}-15\right)\;*\;0.1\right) \end{array}$$

However, I also assume that child mortality risk is a function of maternal marriage age through a second pathway, whereby mothers who marry at older ages have gained greater autonomy, improving control of the household food and monetary budget and access to healthcare, thereby reducing child mortality risk.

I thus assume that child mortality rate (MR) is a composite outcome, linked to both (a) the risk of low birth weight, and (b) the risk of post-natal child mortality. For convenience, I assume that child mortality rate refers to the period before 5 years of age, an outcome reported in numerous national surveys. I calculate MR as follows:


(4) $$\begin{array}{l} \text{MR }=\text{ LBW }*\;\left(\text{k}2-\left(\text{k}3\;*\;0.065\right)\right) \end{array}$$

where LBW is the fraction of births with low birth weight, k_2_ is a constant with value of 0.7, and k_3_ is a further constant that increases linearly with maternal age at marriage, starting at value 0 at age 15 and increasing at a rate of 1 per additional year of age at marriage. These values for k_2_ and k_3_ have been selected to model child mortality risk across a range declining from 70% for AFP of 16 years to 30% at 21 years. I assume that each child is either alive or dead at 5 years, without specifying any particular age-pattern of child mortality rate.

The rate of child undernutrition, defined as the rate of stunting (S) assessed in early childhood, is assumed to develop in proportion to the rate of low birth weight, further exacerbated by adverse effects of early marriage on nutritional care in infancy, but also offset by the potential for catch-up growth after birth. I therefore multiply LBW by a factor of 0.75 to calculate S.

Maternal lifetime reproductive fitness (LRF) is then calculated based on her AFP and her IBI, from which TF is calculated, and her offspring MR; with each of these terms associated as defined above with age at marriage:


(5) $$\begin{array}{l} \text{Maternal LRF }=\text{ TF }*\;\left(1-\text{MR}\right) \end{array}$$

Paternal LRF is calculated from maternal LRF, further accounting for the risk of non-paternity, associated as described above to maternal age at marriage, as follows:


(6) $$\begin{array}{l} \text{Paternal LRF }=\text{ Maternal LRF }*\left(1-\text{PU}\right) \end{array}$$

where PU is the rate of paternity uncertainty as a fraction of 1. I assume that paternity uncertainty increases linearly with increasing age at marriage, from 0 at 15 years to 0.25 at 20 years. Any “lost” paternity is assumed to go to unrelated males. Extra-pair matings clearly contribute to maternal fitness, but the magnitude of any benefit depends on whether her husband invests care and resources in offspring that are not genetically his own.

Using these data, I calculated the optimal age of marriage for a mother in order to maximize her LRF, taking into account the trade-off that earlier marriage promotes higher rates of child mortality, whereas later marriage reduces her overall fertility. I likewise calculated the optimal age of marriage for a father to maximize his fitness, taking into account the same assumptions as for the mother, plus the association between later age at women's marriage and lower paternity confidence. Differences (Δ) in outcome values between the optimal maternal and paternal marriage age were also calculated. Once the optimal outcomes had been identified, I determined the values of the input variables that underlie these outcome values.

Several simulations were then undertaken, in order to predict how changes in individual parameters might affect the optimal age of women's marriage. For each of these scenarios, the optimal age of women's marriage that would maximize maternal and paternal fitness and their LRF was recalculated. In each simulation, other than the specific variables manipulated as described below, all other assumptions and relationships remained constant. The scenarios are as follows:

- To simulate a public health intervention promoting longer inter-birth intervals, this parameter was fixed at 3 years across all categories of marital age.
- To simulate either a public-health intervention that reduced child mortality, or an economic/environmental shock that increased child mortality, I systematically decreased or increased the risk of post-natal mortality, while maintaining its association with the prevalence of low birth weight and the magnitude of female autonomy. I introduced a 10% reduction in child mortality risk from the intervention, and a 20% increase from a shock. These alterations are realistic, given that the majority of children's deaths in low-income settings could be prevented with full implementation of a few simple interventions (79).
- To simulate the consequences of an intervention to promote women's education regardless of the age at which marriage occurs, assumed to promote women's autonomy, I increased the inter-birth interval to 3 years reflecting greater uptake of contraception, and incorporated a 10% reduction in child mortality risk, but I also raised the rate of paternity uncertainty to 0.2 across all categories of marital age. Traditional South Asian societies typically maintain severe sanctions on infidelity, for reasons discussed in detail above. In other societies, however, rates of extra-pair paternity (EPP) can be relatively high. Among Himba pastoralists of Namibia, for example, the rate was 48%, with 70% of couples having at least one EPP child (56). My aim here is to model the effect of infidelity increasing to an intermediate level in association with later age at marriage. It should be noted that men might make efforts to prevent extra-pair matings, even if the actual risk of them happening is lower than assumed.
- To simulate high levels of “family monitoring” of women's behavior in the marital household, across all categories of marital age I fixed the risk of female infidelity at 0.01, and IBI at a low value of 2 years due to lack of access to contraception. In this scenario (A), the mother may not herself have autonomy to take her children for medical care, but I assumed that other members of the marital household would undertake this role in compensation, i.e., they would undertake the same protective tasks that the mother could not perform, resulting in no change in mortality risk compared to the basic model. In a second scenario (B), I assumed that no family member took on this compensatory role, and that as a consequence post-natal child mortality rose by 20%, while also still tracking maternal marriage age. Given the primary role of mothers in both the medical and nutritional care of their children in early life, the impact of the marital household failing to provide inadequate childcare was also assumed to increase the risk of the children being stunted threefold.
- I evaluated how the results of the basic model would change, if maternal mortality were elevated in declining dose-response manner with maternal age at reproduction. Direct evidence for maternal mortality and marriage age is scarce, due to a lack of prospective longitudinal studies in low-income settings. However, early marriage has been associated with several risk factors for maternal mortality (80, 81). Maternal mortality risk (MMR) was assumed to decline at decelerating rate, using the following equation:

(7) $$\begin{array}{l} \text{MMR }=\text{ k}4-\left(\text{k}5\;*\text{ M}\right)\;+\;\left(\text{k}6\;*\;{\text{M}}^{3}\right) \end{array}$$

where M = age at marriage, and values for k_4_, k_5_, and k_6_ are 0.485, 0.033 and 0.000023, respectively. This results in maternal mortality risk varying from 0.070 for marriage at 15 years to 0.016 for marriage at 20 years. Maternal LRF was then recalculated using the following equation:


(8) $$\begin{array}{l} \text{Maternal LRF }=\text{ TF }*\;\left(1-\text{MR}\right)\;*\;\left(1\;-\text{ MMR}\right) \end{array}$$

I assumed that fathers did not remarry if the wife died, hence paternal fitness remained based on maternal fitness, adjusting for paternity uncertainty which in this model has the same association with marriage age as the basic model.

## Results

**Figure 4:**
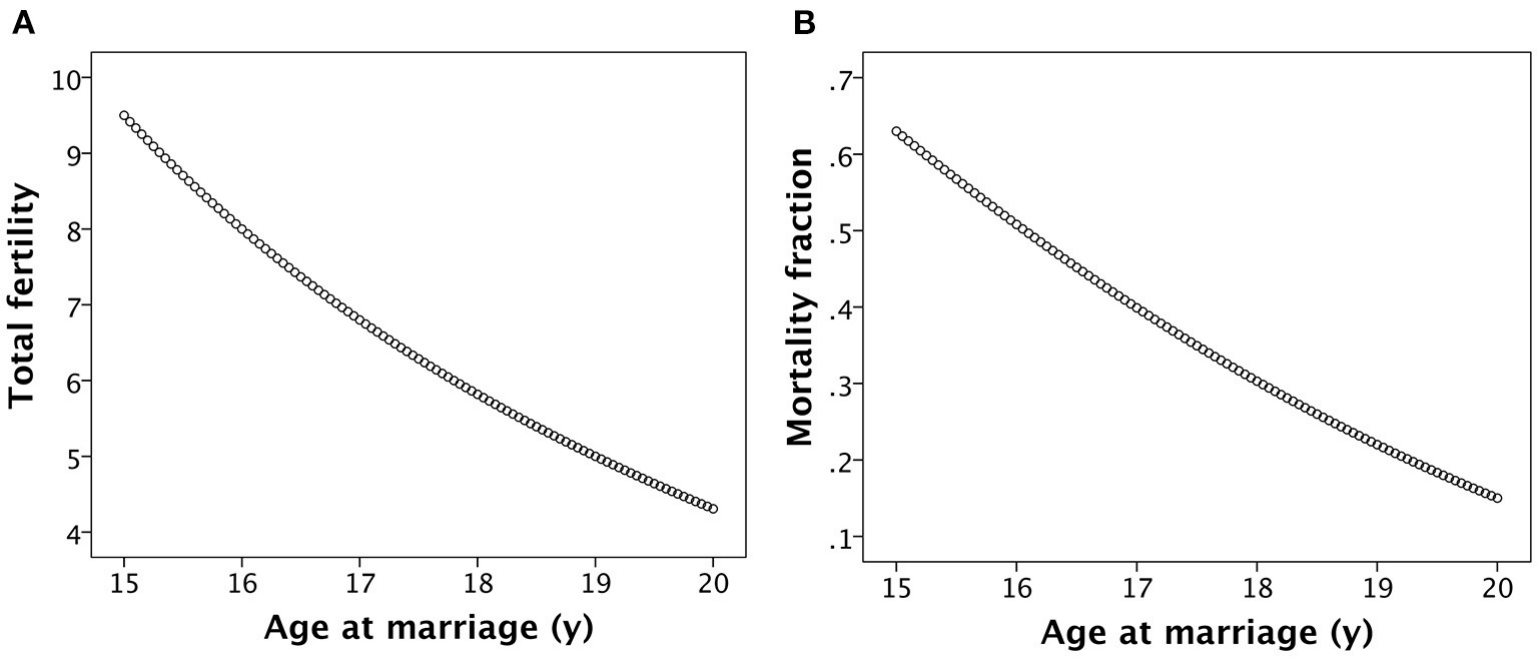
Basic associations of age at women's marriage with **(A)** their lifetime fertility and **(B)** the rate of child mortality, calculated as the fraction surviving by combining the associations illustrated in Figure 2.

The association between age at marriage and total fertility of women up to the age of 35 years is illustrated in Figure 4A. The additive effects of the various assumptions in the model on fertility are strong, such that women marrying at 15 years in this model produce 9.5 offspring by 35 years, whereas women marrying at 20 years produce only 4.2 offspring.

The steady accretion of maternal capital with increasing marital age, mediated in part by the longer inter-birth interval, translates into declining child mortality rate with later age at marriage (Figure 4B). Thus, whilst women marrying younger produce more offspring, they suffer substantially higher rates of both maternal and offspring mortality, whilst women marrying later produce fewer offspring but their offspring are more likely to survive.

**Figure 5:**
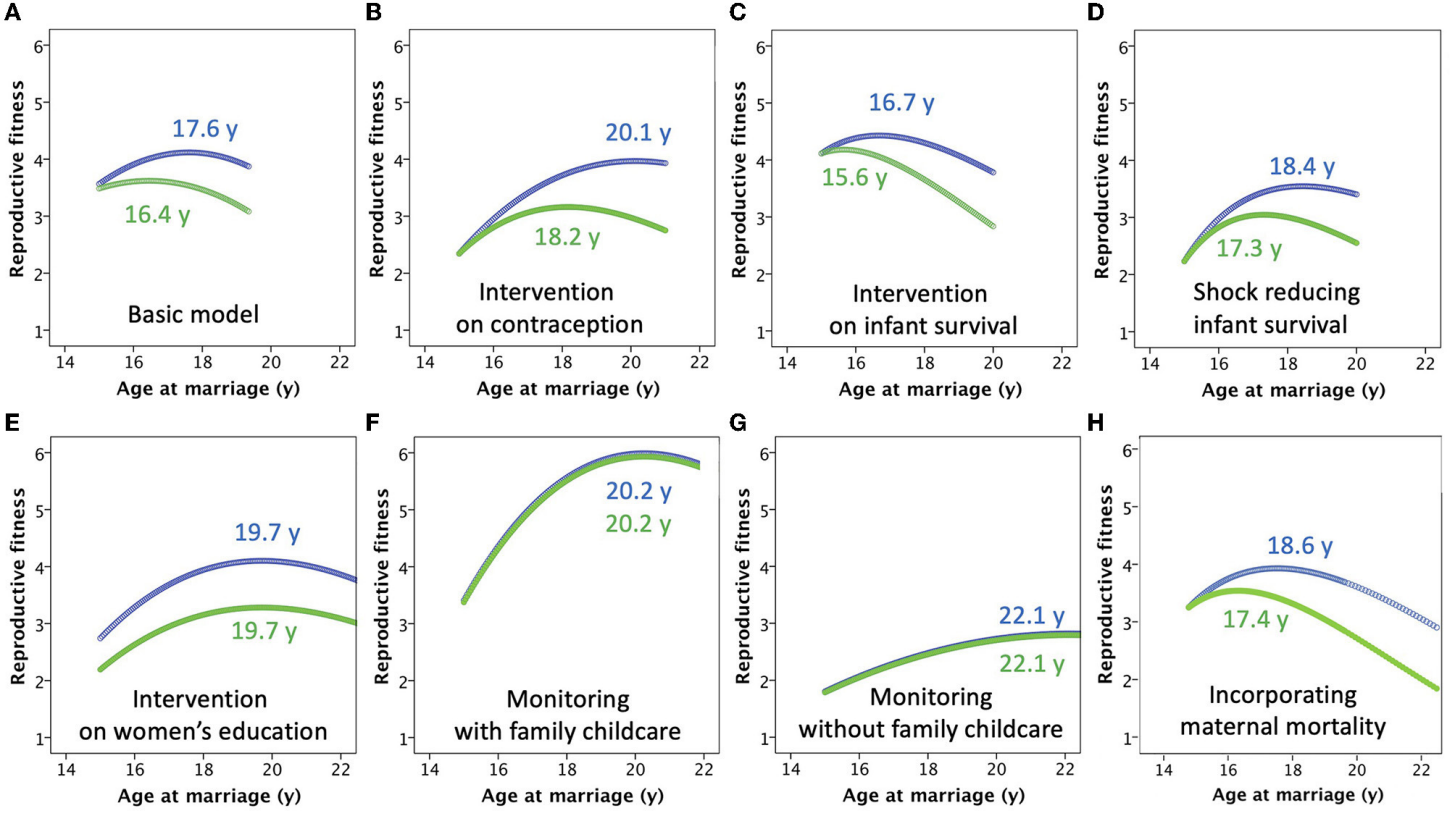
Results of the models, with supporting numeric values given in Table 1. **(A)** The basic model. **(B)** The model incorporating a “contraception” intervention, which standardizes the inter-birth interval at 3 years. **(C)** The model incorporating a public health intervention that reduces child mortality risk by 10%. **(D)** The model incorporating an economic shock that increases child mortality by 20%. **(E)** The model incorporating an intervention to increase women's education, which simultaneously reduces mortality risk, while increasing both the inter-birth interval and paternity uncertainty. **(F)** The model incorporating “monitoring” by the marital household of the mother, which reduces the inter-birth to 2 years and increases child mortality risk, while minimizing paternity uncertainty. The marital household compensates for suppression of maternal autonomy by taking on responsibility for providing medical care for the children **(G)** The model incorporating “monitoring” by the marital household of the mother, similar to the previous scenario but where compensatory support by other household members is not provided. **(H)** The model incorporating a declining risk of maternal mortality with increasing age at marriage, due to later onset of reproduction and longer inter-birth intervals.

Results comparing maternal and paternal fitness for all models are presented together in Figure 5. Numerical values for outcomes (optimal marriage age, lifetime fitness) and underlying values for input variables (child mortality, child malnutrition) that correspond to these outcome values are given in Table 1. Although values for the outcomes are calculated from inputs including risk of child malnutrition and mortality, once the optimal outcomes have been determined it is informative to see the associated underlying levels of child malnutrition and mortality.

**Table 1:** Parameters of models associated with optimal reproductive fitness of each parent.

**Model**	**Optimal marriage**			**Lifetime fitness**			**Child mortality**			**Stunting**		
	**age (years)**			**(offspring)**			**rate (%)**			**prevalence (%)**		
	**Mother**	**Father**	**Δ**	**Mother**	**Father**	**Δ**	**Mother**	**Father**	**Δ**	**Mother**	**Father**	**Δ**
Basic	17.6	16.4	−1.2	4.1	3.6	−0.5	34.0	46.3	12.3	48.0	57.0	9.0
Contraception	20.1	18.2	−1.9	4.0	3.2	−0.8	14.4	28.5	14.1	29.2	43.5	14.3
Public health	16.7	15.6	−1.1	4.4	4.2	−0.2	39.5	49.7	10.2	54.7	63.0	8.3
Economic shock	18.4	17.3	−1.1	3.5	3.0	−0.6	35.3	47.0	11.7	42.0	50.2	8.2
Education	19.7	19.7	0	4.1	3.3	−0.8	14.0	14.0	0	32.0	32.0	0
Monitoring with support	20.2	20.2	0	6.0	5.9	−0.1	19.5	19.5	0	28.5	28.5	0
Monitoring without support	22.1	22.1	0	2.8	2.8	0	52.6	52.6	0	28.5	28.5	0
Maternal mortality added	18.4	17.5	−0.9	4.0	3.6	−0.4	34.0	45.7	10.6	48.0	57.0	6.7

*Optimal marriage age and lifetime fitness are outcomes of the model*.

*Child mortality rate and stunting prevalence are model inputs that vary with marriage age; once the optimal marriage age has been ascertained, the relevant input values can be extracted*.

For the basic model, for which the assumptions are those illustrated in Figures 2, 3, the net effect of these counterbalancing factors is that the optimal age of marriage for maximizing maternal fitness is 17.6 years (with the first offspring produced at 18.6 years), with total fertility of 6.2 offspring but giving LRF of 4.2 offspring (Figure 5A). For the father, genetic fitness is maximized when women marry at 16.4 years and have fertility of 7.5 offspring, though due to greater mortality and non-paternity male LRF is 3.6 offspring. There is thus a difference in the optimal age of women's marriage between the two parental strategies of 1.2 years, which emerges because of increasing paternity uncertainty with later marriage age. Though optimal for the fitness of one or other parent, in each case rates are high for both child mortality (34.0% for maternal optimum, 46.3% for paternal) and stunting (48.0% for maternal optimum, 57.0% for paternal). The rates of child mortality and stunting are therefore 12.3 and 9.0% greater, respectively, if the age at marriage is at the paternal rather than the maternal optimum.

If the inter-birth interval is fixed at 3 years regardless of marriage age, due to a family planning intervention promoting contraception, LRF declines for mothers marrying earlier, as they have lower capacity to counter their high risk of offspring mortality by producing numerous offspring through their reproductive career (Figure 5B). The optimal age at marriage for women shifts forwards to 20.1 years, giving total fertility of 4.6 offspring and LRF of 4.0 offspring. However, this benefit impacts less on fathers, as they do not share the benefit from lower infant mortality, hence their optimal marriage age for women is 18.2 years, now 1.9 years lower than that which maximizes maternal fitness, giving mothers fertility of 5.3 offspring and fathers LRF of 3.2 offspring. In this scenario, the longer inter-birth interval decreases mortality and stunting rates compared to the basic model regardless of whether the marriage age is optimal for mothers or fathers, however the rates of child mortality and stunting remain greater by 14.1 and 14.3%, respectively, if the age at marriage is at the paternal rather than the maternal optimum.

Simulating a public health intervention to reduce child mortality by 10%, with no change in fertility, the effect is to increase the number of surviving offspring for each parent (Figure 5C). However, these benefits occur disproportionately in women marrying younger, who previously suffered the greatest levels of child mortality. To capitalize on this benefit, the optimal age for marriage to maximize maternal fitness therefore shifts earlier, to 16.7 years, giving LRF of 4.4 offspring, slightly higher than in the basic model. A similar decline occurs in the optimal age of marriage to maximize paternal fitness (15.6 years), giving LRF of 4.2 offspring, with the difference in optimal age between the two parents reduced to 1.1 years. Non-intuitively, the increase in the LRF of each parent derives from starting reproduction earlier and increasing fertility, so that although the age-standardized risk of child mortality is reduced, the overall proportion of offspring dying is higher than in the basic model (37.9% for maternal optimum, 49.6% for paternal), and rates of child malnutrition are also very high (54.7% for maternal optimum, 63.0% for paternal).

Conversely, simulating an economic or environmental shock that increases child mortality by 20%, the optimal age at marriage shifts forwards 18.4 years to maximize maternal fitness and 17.3 years for paternal fitness, giving LRF of 3.5 and 3 offspring, respectively (Figure 5D). For both parents, a later age at women's marriage is favored to counter the very high rates of child mortality amongst early marrying mothers. The difference in optimal age at marriage between the two parents remains, because delaying the age at marriage also increases paternity uncertainty. The later onset of reproduction leads to lower LRF than in the basic model (mothers 3.5 and fathers 3.0 offspring), but similar levels of child mortality (35.3% for maternal optimum, 47.0% for paternal), and lower rates of stunting (42.0% for maternal optimum, 50.2% for paternal). Overall, an external spike in mortality risk is therefore expected to delay marriage and reproduction, overall reducing the rate of malnutrition.

An intervention promoting women's education reduces child mortality risk, which benefits both parents, but it also increases the opportunity for contraception such that IBI increases, and reduces paternity certainty due to greater women's autonomy. Mothers gain from higher rates of offspring survival associated with later marriage, while fathers are less able to compensate for the high early mortality associated with early marriage through greater fertility. Thus the two parents now converge on the same optimal age of marriage, of 19.7 years (Figure 5E), corresponding to fertility of 4.8 offspring. For mothers, LRF (4.1) is very similar to the basic model, showing how they have benefitted from the combination of better child survival and control of fertility, however for fathers LRF (3.3) is lower than the basic model due to higher rates of paternity uncertainty. However, rates of child mortality (14.0%) and child stunting (32.0%) are equally low for both parents.

Familial efforts to increase the “monitoring” of maternal behavior, reducing the risk of infidelity to 0.01, also result in the two parents converging on the same optimal age of women's marriage (an automatic outcome given the basis of the model), but at a later age compared to the basic model. Minimizing paternity uncertainty, there is no longer any payoff for males preferring an earlier marital age than their wives, while the shorter IBI regardless of the age at marriage provides high fitness benefits later in the reproductive career, reducing the benefits of starting reproduction at an early age. In the first scenario modeled, where members of the marital household compensate for suppressing the mother's autonomy by taking on responsibility for childcare, fertility is high at 6.9 offspring, and the optimal age at marriage for both parents is 20.2 years (Figure 5F). LRF is relatively high for both mothers and fathers, at 6.0 and 5.9 offspring, respectively. The combination of delaying the onset of reproduction and the provision of childcare results in lower levels of child mortality (19.5%) and stunting (28.5%), compared to the basic model.

However, if the marital household does not compensate for the suppression of maternal autonomy, then the fitness of both parents is reduced despite total fertility of 5.9 offspring. In order to avoid very high rates of child mortality early in the reproductive career, the optimal age at marriage of both parents is 22.1 years (Figure 5G). However, this later onset of reproduction, combined with high mortality rates, leads to LRF of only 2.8 offspring for both father and mother. For both parents, child mortality rate is 52.6%, and the rate of stunting is 42.8%. In this scenario, parental tension over infidelity is absent, but the cost to maternal autonomy and her ability to care for her offspring is so great that the fitness of both parents suffers.

If maternal mortality rate was assumed to decline exponentially from 7% among mothers who married at 15 years, to 2% among those who married at 20 years, then the optimal age at marriage shifts to the right for both parents, being 18.6 years for mothers and 17.4 years for fathers (Figure 5H), compared to values of 17.6 and 16.4, respectively in the basic model. However, there was very little change in lifetime fitness of child malnutrition and mortality (Table 1).

## Discussion

**Figure 6:**
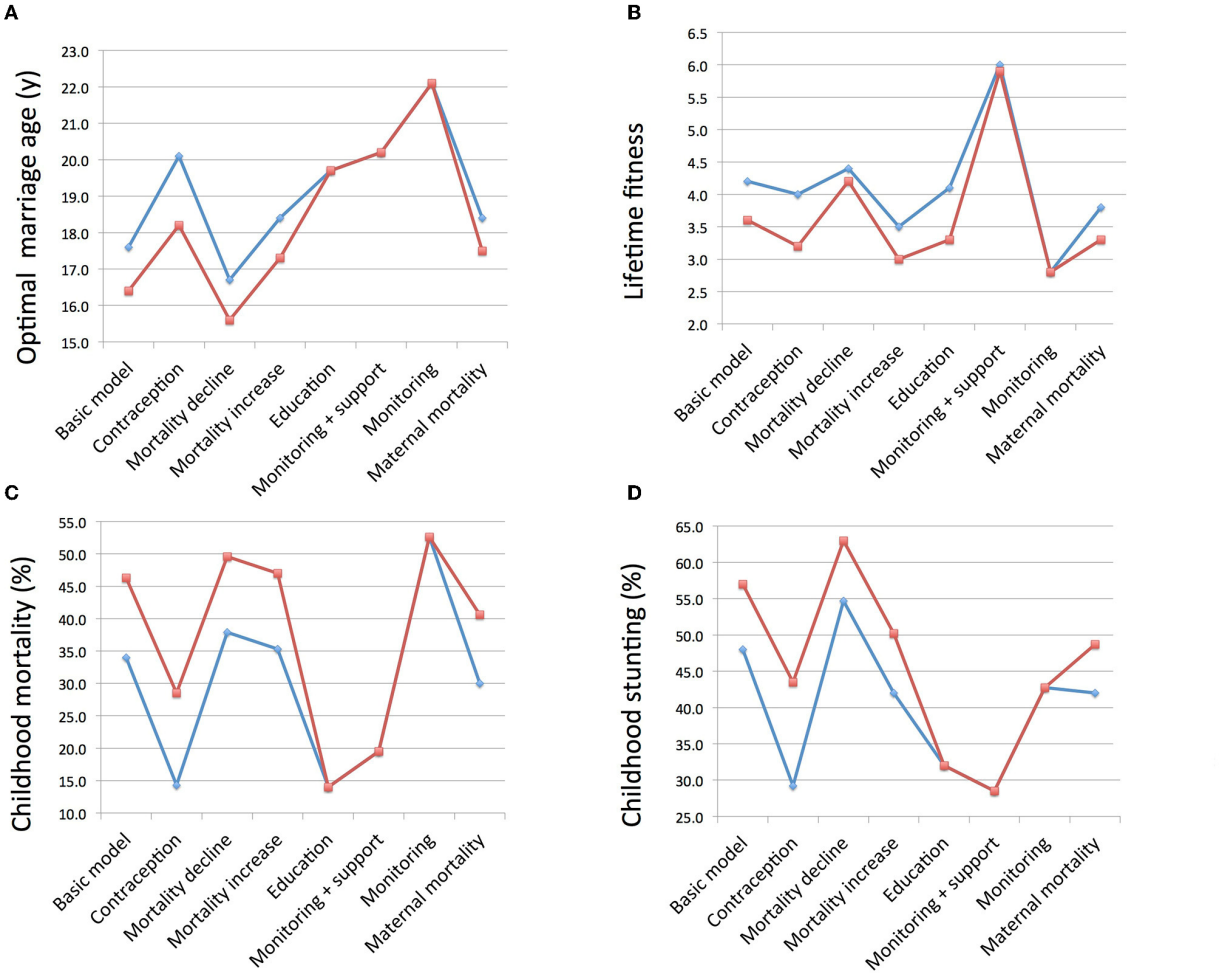
Summary of main findings across all the models, showing values corresponding to the optimal age at marriage for women of mothers (blue) and fathers (red). **(A)** Optimal age at marriage. **(B)** Lifetime reproductive fitness calculated as the number of surviving offspring. **(C)** Rate of child mortality relative to all children born. **(D)** Rate of child undernutrition assessed in terms of stunting.

This study explored how each parent might maximize the number of surviving offspring produced, taking into account how variability in maternal marriage age and the schedule of her reproductive career is associated with each of fertility rate, child survival and the level of paternity uncertainty. A summary of the main results from the different models is given in Figure 6, showing that in the majority of specific models, the optimal age for women at marriage differs according to whether maternal or paternal fitness is maximized, and this has implications for the number of viable offspring produced, and the rates of child mortality and undernutrition. Under some circumstances, the parents converge on more similar optimal values for marriage age, whereas under other conditions they diverge, indicating greater intra-family tension from a theoretical standpoint.

Although women's age at marriage was treated as the key variable, in societies where early marriage is common women themselves tend to have little or no control over their marriage age. Rather, the timing represents a family decision, made in the interests of other family members in both the natal and marital households. However, given that in patriarchal agricultural societies a woman's main life pathway is marriage, and the main social purpose in life ascribed to her is to bear several children, in particular sons, a young woman's natal family have little choice but to go with men's preferred optimal marriage age. Since the model assumes that the first offspring is produced one age after the woman's marriage, I identified the optimum age at which a woman should be married (a cultural behavior) if the only aim were to maximize either paternal and maternal fitness. From an evolutionary perspective both natal and marital households benefit from promoting maternal fitness, but there may be associated costs to health (e.g., higher levels of child stunting) or human capital (e.g., lower levels of maternal education).

Before reviewing the model findings in detail, it should be emphasized that early marriage is already widely understood to inflict a range penalties on women (15, 17), many of which propagate to their offspring (13, 37). The aim in applying an evolutionary framework is *not to justify* early marriage, but rather to examine why males may favor the practice, despite the associated risk of mortality for their wives and own offspring. In these models, the outcome of different marriage strategies of each sex is being evaluated relative to others of the same sex. Therefore, men may maximize their fitness relative to other men, through strategies that inflict substantial penalties on women. The key finding is that under most circumstances, men maximize their fitness through earlier marriage than is optimal for female fitness, even though this is associated not only with women's disempowerment but also with higher rates of offspring mortality and undernutrition. This modeling ignores any additional fitness pay-offs that might accrue to the husband through extra-pair matings, however a key point is that he would have minimal opportunity to invest economic resources in such offspring, given social norms over women's fidelity. Improved understanding of how this pattern then varies in association with other factors may help identify new opportunities to reduce the practice of early marriage.

The model assumes that if women's marriage is “too” early, the lack of maternal autonomy and capital translates into high levels of offspring mortality. On the other hand, if marriage is delayed, the greater level of maternal autonomy over reproduction increases the inter-birth interval and reduces fertility. In the basic model, these counterbalancing associations gave an optimal marriage age for maternal fitness of 17.6 years.

However, according to the assumptions used, this marital age has already allowed the mother to acquire some autonomy, allowing her to lengthen the inter-birth interval while also reducing paternity certainty. To counter these effects, a man would maximize his own fitness, relative to other men whose wives were married earlier or later, if his wife was aged 16.4 years at marriage. Although this younger marital age is associated with higher rates of child mortality and stunting, these penalties are more than offset from the perspective of paternal fitness by the longer reproductive career of the wife, the shorter inter-birth intervals, and the higher level of paternity certainty.

Thus, the basic model immediately identifies a fundamental tension between the two parents over the optimal age at marriage, consistent with the principles of reproductive conflict over fertility, mortality risk and paternity certainty. If there were no association between maternal age at marriage and paternity uncertainty, the contrast in optimal marriage age (and hence the parental conflict of interest) between the two parents would entirely disappears, and all that would remain would be uniform differences between the parents in their fitness, determined by whatever “fixed” level of paternity uncertainty was incorporated into the model.

I then explored how several potential simulated “interventions” could impact this parental tension, changing the optimal age of women's marriage for each parent through differential effects on child survival and mortality, associated with changes in inter-birth interval and maternal autonomy, as well as the level of paternity uncertainty. For example, lengthening the birth interval, representing a “family planning intervention”, increased the optimum age of marriage for both parents. This shift occurs because it is no longer possible for early-marrying women to counter-balance their high rates of child mortality by producing offspring fast. This impacts both paternal and maternal fitness, and results in a divergence between the two parents regarding the optimal age of marriage, due to at a greater cost to paternal than maternal fitness, because of the increased paternity uncertainty.

The model paid no attention to a range of factors known to influence decisions over marriage age, or the consequences of early/later marriage. These include socio-economic factors, including markers of poverty and the capacity to make dowry payments in the natal household, and factors relating to food and economic security and wealth in the marital household, which may further relate to paternal education. In effect, the model treats all these variables as constants, however others have incorporated them in models, for example of marriage as a social contract (82) or game theory models of dowry offers (83).

The model was developed specifically for low-income patrilineal patrilocal populations, where maximizing fertility is key to wealth and food security through subsistence agriculture. The model assumes that child mortality is a major factor, and hence is unlikely to be generalizable to other economic scenarios, where child mortality is low and high fertility has different economic implications.

Some of the results of the model may appear counter-intuitive. For example, in general child mortality and early marriage are correlated, hence over time we would expect that societies with falling child mortality would also demonstrate later marriage. Conversely, Figure 5C indicates that improved offspring survival was associated with earlier optimal marriage age of both parents. This apparent contradiction is explained by the fact that the model varies only a single variable at any time, holding others constant, whereas in real life, a suite of variables may all change in combination. The implication is that simply changing infant mortality would not delay marriage, if the only goal were to maximize parental fitness.

Likewise, the latest optimal ages for marriage age emerged from models that incorporated family monitoring, a practice that is clearly contrary to women's autonomy. Since empirical studies have widely linked such family monitoring with early marriage and a preference for large family size (84), why does the model predict that fitness is actually maximized at later age at marriage? This contradiction may potentially be resolved through the penalty of maternal mortality, which was not addressed in this version of the model. Using data from 97 countries, a simulation suggested that a 10% increase in the rate of early marriage (defined as <18 years) would increase the maternal mortality ratio by 70% (85). Thus, families that combine early marriage with the pressure to produce offspring regularly may only maximize the fitness of the male if they replace women who die through maternal mortality, itself directly related to lack of autonomy. This does not indicate that family monitoring is a desirable intervention to delay marriage. Rather, from the perspectives of women's rights and public health, women should be empowered to gain control over their fertility.

The model also assumed large differences in risks or benefits associated with varying age at marriage. This approach was used in order to help visualize potential consequences, and further work could test more nuanced associations, for example incorporating weaker gradients, or specifying non-linear associations for the traits displayed in Figure 3. My aim here was simply to highlight the potential for conflict between males and females over the optimal age of marriage for fitness, and to further show that the values respond to ecological factors. Moreover, while the values selected were inevitably arbitrary, all aspects of the model are broadly supported by literature.

This suggestion is relevant to the final iteration of the model, which found that an increased risk of maternal mortality among women who marry early unsurprisingly favors a later age at marriage for mothers, though the difference compared to the basic model was small. Since the model assumed that the husband does not replace his wife, adding maternal mortality to the model results in husbands also favoring later marriage age. In practice, given the capacity of men to remarry a younger woman and maximize fitness across successive wives, maternal mortality does not on its own provide a disincentive to marrying wives of young age. Precisely because of the complexity of modeling the consequences of husbands having more than one wife, I did not incorporate maternal mortality risk in the basic model, and this aspect would benefit from further work with more dynamic modeling.

Overall, this simple model shows that the optimum age at marriage is sensitive to a number of variables. Using predictions based on published evidence for associations between maternal age at marriage and maternal or child phenotype, the model allowed exploration of intra-familial tension over maternal fertility and investment of “nutritional capital” in offspring, and of how varying age at marriage fundamentally influences the payoffs for different kin and household members.

The model helps understand an apparent paradox: that males favor early marriage of their wives, even though this is associated with lower rates of maternal education and higher levels of child malnutrition and mortality. The husband gains fitness pay-offs from reducing his wife's autonomy, even though his own children on average suffer a nutritional penalty that worsens their survival chances. From the male's perspective, this penalty is more than offset by the greater fertility rate of women married early. This scenario helps explain why age at marriage may not shift markedly even as opportunities for female education increase, if education does not genuinely improve women's autonomy and control over reproduction. For example, recent studies from South Asia indicate that where education has until recently been scarce, parents primarily educate women in order to improve their chances in marriage markets, which impacts the parents' social status, rather than to empower women (17). In such societies, women with high levels of education may not be considered ideal brides as they may be less amenable to control by other members of the marital household.

The model has several limitations. The assumptions that were incorporated represent a simplification of the full range of factors, and the relationships were assumed to have linear form, although in practice more complex functions may apply. Changes in child phenotype across successive offspring of individual women were not addressed. The numerical values that were extracted as outcomes are not “real” values, rather the modeling parameters were selected for illustrative purposes, simply to show how outcome values may be increased or decreased according to the assumptions involved. Moreover, while the rate of child undernutrition was extracted as an outcome, it was also (expressed as the risk of low birth weight) incorporated into the model. However, the aim was to show how, given such specified associations, variability in the rate of child undernutrition is to be expected if the age at women's marriage also varies. I did not address variability in men's marital age, as there is less pressure from an evolutionary perspective for men to marry early, and in societies where early women's marriage is common, there can be wide discrepancies in age across husband-wife dyads. I also did not consider how the sex ratio of offspring might impact subsequent fertility decisions and child outcomes, as families often place a high value on sons (86, 87).

More broadly, the model includes only two generations, whereas it is increasingly appreciated that human reproduction is essentially a three-generation process. In many populations, as successive generations replace each other, a disempowered wife becomes a more empowered mother-in-law, who now has a vested interest in supporting her sons' behavior. In patrilocal societies, a mother's daughters are less accessible to her, hence her reproductive interests now align more with those of her son. The mother-in-law might therefore play a particular role in “monitoring” of the wife's behavior, an example of the general monitoring scenario as explored in this model. However, as wives reach post-reproductive age, the value of the mother-in-law is also expected to decline. This suggests that individual women will be valued very differently within the household depending on the stage of their reproductive career, and that mothers-in-law might be at risk of malnutrition in older age as they are “replaced” by a younger post-reproductive female.

In conclusion, this study used a simple model to illustrate how variable associations of age at marriage with markers of maternal biology and autonomy are expected to create intra-family tension in the optimal age for women's marriage, if the only aim is to maximize fitness. From this perspective, males may benefit from behavioral strategies that impair the health not only of their wife, but also of their own individual offspring, and hence actively prefer young brides. This tension is expected to be altered by different forms of environmental change or public health interventions, which impact the various parameters in the model in different ways. An education intervention would have the greatest benefit for women and children, but according to the assumptions in the model would do so at a potential cost to male fitness. This may help understand societal norms that counter efforts to reduce the prevalence of early marriage, and hence why early marriage may remain common in many populations. In contemporary human populations, fitness is undoubtedly not the only outcome maximized, but fitness-enhancing behavior is likely to remain relevant, especially in societies where farm productivity is closely associated with family labor. The tension is expected to be exacerbated in patriarchal patrilocal societies, where the transfer of material property down the male line will only benefit the husband's genes if the offspring of his wife are indeed his own.

## Data Availability Statement

The original contributions presented in the study are included in the article/supplementary material, further inquiries can be directed to the corresponding author/s.

## Author Contributions

The author confirms being the sole contributor of this work and has approved it for publication.

## Funding

This work was undertaken as part of a project on early marriage funded by the Leverhulme Trust (RPG-2017-264).

## Conflict of Interest

The author declares that the research was conducted in the absence of any commercial or financial relationships that could be construed as a potential conflict of interest.

## Publisher's Note

All claims expressed in this article are solely those of the authors and do not necessarily represent those of their affiliated organizations, or those of the publisher, the editors and the reviewers. Any product that may be evaluated in this article, or claim that may be made by its manufacturer, is not guaranteed or endorsed by the publisher.
